# Chest pain out-of-hours – an interview study of primary care physicians’ diagnostic approach, tolerance of risk and attitudes to hospital admission

**DOI:** 10.1186/s12875-014-0207-4

**Published:** 2014-12-21

**Authors:** Robert Anders Burman, Erik Zakariassen, Steinar Hunskaar

**Affiliations:** National Centre for Emergency Primary Health Care, Uni Research Health, Kalfarveien 31, 5018 Bergen, Norway; Department of Global Public Health and Primary Care, University of Bergen, Post box 7804, 5020 Bergen, Norway; Department of Research, Norwegian Air Ambulance Foundation, Post box 94, 1441 Drøbak, Norway

**Keywords:** Chest pain, Primary care, Out-of-hours, Diagnostic approach, Clinical decision rules, Tolerance of risk

## Abstract

**Background:**

Acute chest pain constitutes a considerable diagnostic challenge outside hospitals. This will often lead to uncertainty in choosing the right management, and the physicians’ approach may be influenced by their knowledge of diagnostic measures and their tolerance of risk. The aim of this study was to investigate primary care physicians’ diagnostic approach, tolerance of risk and attitudes to hospital admission in patients with acute chest pain out-of-hours in Norwegian primary care.

**Methods:**

Data were registered prospectively from four Norwegian casualty clinics. Data from structured telephone interviews with 100 physicians shortly after a consultation with a patient presenting at the casualty clinic with “chest pain” were analysed. Tolerance of risk was measured by the Pearson Risk Scale and the Tolerance of Risk Scale, the latter developed for this study.

**Results:**

“Patient history and symptoms” was considered the most important, and “negative ECG” and “effect of sublingual nitroglycerine” the least important aspects in the diagnostic approach. There were no significant differences in length of experience or gender when testing “risk avoiders” against the rest. Almost all physicians felt that their risk assessment out-of-hours was reasonably good, and felt reasonably safe, but only 50% agreed with the statement “I don’t worry about my decisions after I’ve made them”. Concerning chest pain patients only, 51% of the physicians were worried about complaints being made about them, 75% agreed that admitting someone to hospital put patients in danger of being “over-tested”, and 51% were more likely to admit the patient if the patient herself wanted to be admitted.

**Conclusions:**

Physicians working out-of-hours showed considerable differences in their diagnostic approach, and not all physicians diagnose patients with chest pain according to current guidelines and evidence. Continuous medical education must focus on the diagnostic approach in patients with chest pain in primary care and empowerment of physicians through training and emphasis on risk assessment and “tolerance of risk”.

## Background

Acute chest pain still constitutes a considerable diagnostic challenge outside hospitals, especially when it comes to separating potential life-threatening illnesses (e.g. acute coronary syndrome) from less serious conditions (e.g. thoracic myalgia or dyspepsia) [1-4]. Attempts have been made to develop valid clinical decision rules for patients with acute chest pain in primary care, but extensive research have shown that determining the cause of chest pain, without cardiac markers (ie. troponin) and more advanced diagnostic tools, is a difficult task [5-9]. It is still unclear if clinical decision rules are suitable for such a complex diagnostic situation.

In Norway, many patients with acute chest pain choose to contact their general practitioner directly, or the local casualty clinic out-of-hours, instead of calling the national emergency three digits number “113”. Previous research has shown that “chest pain” is one of the most common complaints in out-of-hours primary care [10], and we have recently published a paper describing the challenges in managing chest pain outside hospitals [11].

Challenging diagnostics will often lead to uncertainty in choosing the right treatment and level of care for the patient. In primary care, especially the decision to admit a patient with chest pain to a hospital or not can be demanding. Deciding the appropriate management of patients with chest pain, including the decision to admit urgently to a hospital or not, may also be influenced by the physician’s tolerance of risk, and the preferences of both the patient himself and his family. Previous studies have indicated a correlation between physicians’ “tolerance of risk” and admission rates, both for patients in general and patients with chest pain specifically [12-15].

There exists only scarce literature about primary care physicians’ attitudes to admitting patients with chest pain to a hospital. The aim of this study was to investigate primary care physicians’ diagnostic approach, tolerance of risk and attitudes to hospital admission in patients with acute chest pain out-of-hours in Norwegian primary care.

## Methods

Four Norwegian casualty clinics were chosen for cooperation and collection of data, according to strategic sampling. The casualty clinics cover both rural, suburban and urban districts, and include both larger and smaller clinics. Data were collected prospectively from February to July 2012.

The analysed data consist of structured telephone interviews with 100 physicians (each physician interviewed only once) shortly after a consultation with a patient meeting the inclusion criteria. Registration of patients continued until 100 unique physicians with 100 corresponding patients had been included. All patients with “chest pain” or equivalent symptoms as their main symptom, independent of the probable cause of complaint, were registered by nurses at the four casualty clinics. Equivalent symptoms included “tightness in chest”, “retrosternal pain” and “chest discomfort”. Patients with symptoms clearly suggestive of mastitis were excluded. If a physician could not be reached by telephone, and interviewed, within 2 days after the consultation, he or she was excluded from participation, in order to reduce recall bias. The interviewer was a general practitioner with experience in out-of-hours work (author RAB).

The questionnaire used in the telephone interview was divided in to two parts, where the first part consisted of questions related to the patient they just had treated, including “level of response”, diagnostic measures (use of ECG and laboratory analyses), severity of illness, appraisal of most probable cause of symptoms and choice of treatment and level of care.

The results from the first part of the questionnaire, and a more detailed description of the methods of the study, are described elsewhere in a recently published paper [11]. Analyses showed that the study population (n = 100) did not differ from all registered chest pain patients (n = 832) in any of the variables stated, except mean age, the study patients were about 5 years younger [11].

Analyses from part two of the questionnaire are presented in this article. This part of the questionnaire focused on the individual physician’s approach to diagnosing patients with chest pain, the physician’s “tolerance of risk”, and attitudes to hospital admission. Diagnostic approach was measured using a five-point Likert scale where the physicians graded the importance of different aspects of the diagnostic process.

“Tolerance of risk” was measured using the Pearson Risk Scale, and a new Tolerance of Risk Scale, developed for this study.

### Pearson risk scale

The Pearson Risk Scale was developed for triage decisions in patients with chest pain [15]. This scale consists of six items with questions answered along a six-point Likert scale from “strongly agree” to “strongly disagree” (Table 1). The scale divides physicians into one of three categories based on summation of the scores; high scorers (“risk-seeking”) scored one standard deviation or more above the mean, middle-scorers scored midrange, and low scorers scored more than one standard deviation below the mean (“risk-avoiders”).

**Table 1:** Pearson risk scale*- Physician risk attitudes

1.	I enjoy taking risks
2.	I try to avoid situations that have uncertain outcomes
3.	Taking risks does not bother me if the gains involved are high
4.	I consider security an important element in every aspect of my life
5.	People have told me that I seem to enjoy taking chances
6.	I rarely, if ever, take risks when there is another alternative

*All questions were asked on a six-point Likert scale from "strongly agree to strongly disagree".

### Tolerance of risk scale

To develop the Tolerance of Risk Scale, we used the seven first items of a questionnaire from a previously published article (Ingram-questionnaire) [12], slightly adapted to a Norwegian out-of-hours-setting. This questionnaire consists of statements where the physicians should select the appropriate level of agreement according to a five-point Likert scale from “agree strongly” to “disagree strongly”. Furthermore, we used a similar approach to how the Pearson Risk Scale was constructed, dividing the physicians into one of three “risk groups”, naming it the “Tolerance of Risk Scale”.

The Pearson Risk Scale measures physician “risk attitudes” in general, while the newly developed Tolerance of Risk Scale specifically measures “risk attitudes” working in an out-of-hours-setting.

### Attitudes to hospital admission

Attitudes to hospital admission were measured using 15 items from three dimensions (B - D) of the Ingram-questionnaire [12].

#### Statistics

IBM Statistical Package for the Social Sciences (IBM SPSS version 20) was used for statistical analyses. Standard univariate statistics were used to describe the material, including mean and median. Mann–Whitney U test was used for comparison between the items from the Ingram-questionnaire and the Pearson Risk Scale. For other comparisons Chi-Square tests were used. A P-value of < 0.05 was considered statistically significant.

#### Ethics

The study was given approval by the Regional Committee for Medical and Health Research Ethics (REC West) before inclusion started (Reference number 2010/1499-10).

## Results

The four participating casualty clinics registered a total of 832 patients with chest pain as their main symptom, of which the first 100 unique patient and physician pairs, with completed structured telephone interviews, were included in the study.

The included patients’ (n = 100) age ranged from 18 to 92 years (median age 46 years), 58% males with a median age of 45 years, and 42% females with median age 51 years. The study included 60 male physicians and 40 female physicians. GPs constituted 67%, the rest were interns in general practice (11%) or hospital-based physicians (22%).

Table 2 describes the physicians’ approach to diagnosing patients with chest pain by registering the selected importance of different aspects of the diagnostic process. 99% believed that the patient’s symptoms and history was fairly (19%) or very important (80%) (mean 4.8/5 on Likert scale), while all of the physicians stated that a “positive” ECG-finding was fairly (10%) or very important (90%) (mean 4.9). “Negative” ECG-findings (mean 2.8) and effect of sublingual nitro-glycerine (mean 3.0) were considered to be the least important aspects.

**Table 2:** **Physicians’ appraisal of the importance of different aspects of the diagnostic process along a five-point Likert scale (n = 100)**

		**Degree of importance**				
**Aspects of the diagnostic process**	**Very important**	**Fairly important**	**Neither important nor unimportant**	**A little important**	**Very little important**	**Mean value**
	**(5)**	**(4)**	**(3)**	**(2)**	**(1)**	
Patient's symptoms/history	80	19	1	0	0	4.8
"Negative" ECG findings	3	25	23	46	3	2.8
Effect of sublingual nitroglycerine	5	36	22	29	8	3.0
Chest wall tenderness	3	44	26	25	2	3.2
"Positive" ECG findings	90	10	0	0	0	4.9
Clinical examination	22	50	17	11	0	3.8

Analytic value in brackets.

**Figure 1:**
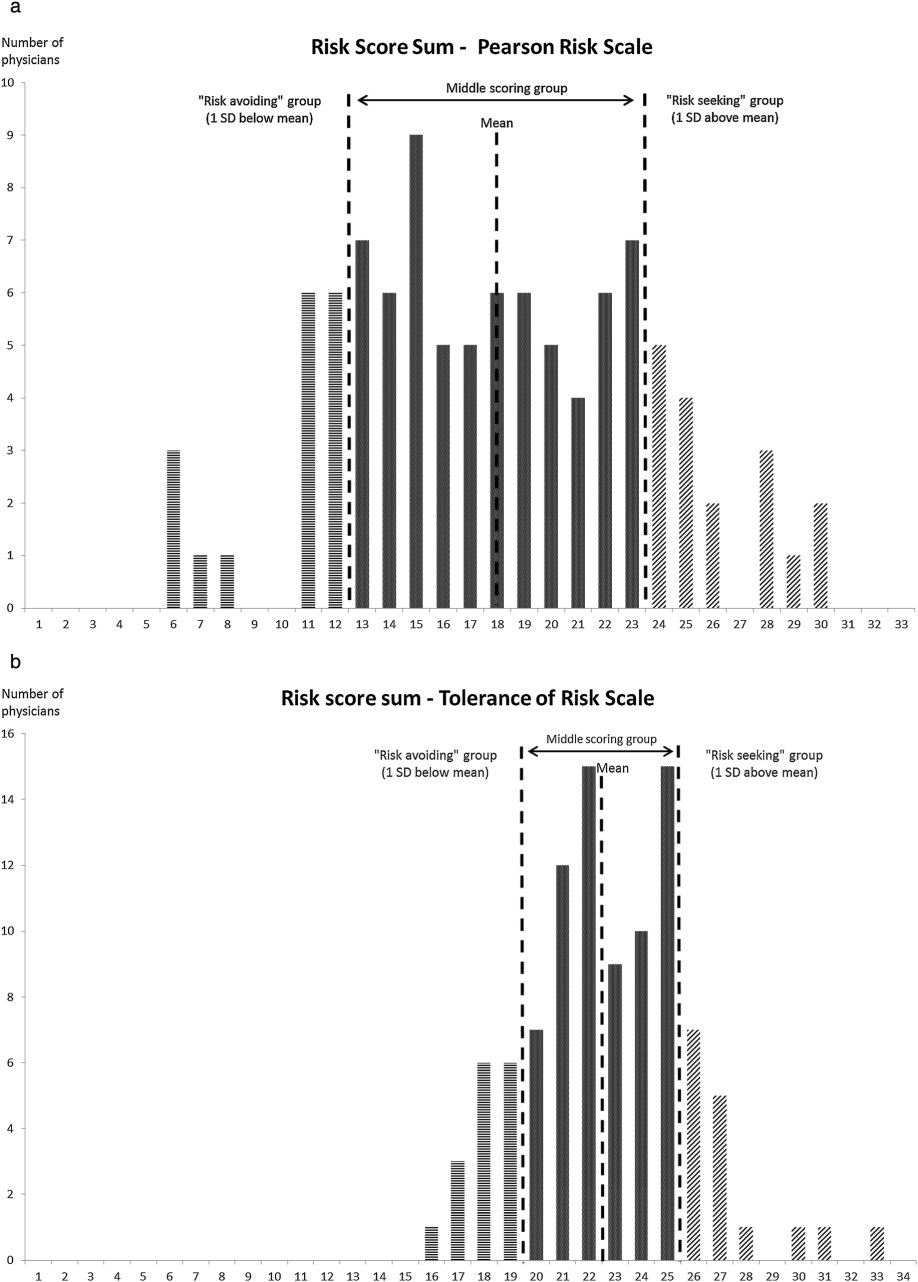
**Risk score sums, dividing the physicians into one of the three groups. a**. Pearson risk scale **b**. tolerance of risk scale.

Figure 1a and b show the risk score sums from the Pearson Risk Scale (Figure 1a) and Tolerance of Risk Scale (Figure 1b). Both scales divide the physicians into three groups; “risk-avoiding”, “middle-scorers” and “risk-seeking”.

Table 3 presents “physician risk attitudes” derived from the Pearson Risk Scale. There was no significant difference in the length of work experience between male and female physicians (p = 0.072). The “middle-scoring” group constituted two thirds (66 of 100), while the groups “risk-avoiders” and “risk-seekers” were equally divided with 17 physicians each. When analysing “risk-avoiders” against the rest, we found no significant differences in length of experience (p = 0.155) or gender (p = 0.913). Analysing “risk-avoiders” against the rest using the Tolerance of Risk scale also showed no significant differences (length of experience p = 0.085; gender p = 0.148).

**Table 3:** **Physicians’ risk attitudes divided in to three groups, by gender and length of work experience**

	**Physicians’ risk attitudes – Pearson risk scale**			
	**Risk-avoiding**	**Middle-scoring**	**Risk-seekers**	**Total**
Male physicians				
Experience 0–5 years	5	17	3	25
Experience more than 5 years	5	25	5	35
Total	10	42	8	60
Female physicians				
Experience 0–5 years	6	11	7	24
Experience more than 5 years	1	13	2	16
Total	7	24	9	40
Total, all physicians	17	66	17	100

Table 4 describes the physicians’ tolerance of risk and uncertainty (dimension A) and concerned all patients out-of-hours. The strongest agreement in dimension A was found in the statement “I think my risk assessment is reasonably good, and I’m reasonably safe”, in which 94% agreed to the statement (67% a little; 27% strongly; mean 4.2). We found the weakest agreement in the statement “I don’t worry about my decisions after I’ve made them”, 46% disagreed (5% strongly; 41% a little), while 50 % agreed (42% a little; 8% strongly).

**Table 4:** **Tolerance of risk and uncertainty, dimension A**

	**Level of agreement**					
	**Agree strongly**	**Agree a little**	**Neither agree nor disagree**	**Disagree a little**	**Disagree strongly**	**Mean value**
	**(5)**	**(4)**	**(3)**	**(2)**	**(1)**	
Tolerance of risk and uncertainty – all patients out-of-hours (OOH)*						
1. When it comes to OOH-medicine I’m quite cautious	13	51	12	22	2	3.5
2. As an OOH-physician you think that you can deal with most things most of the time	18	63	6	11	2	3.8
3. I think my risk assessment is reasonably good, and I’m reasonably safe	27	67	4	2	0	4.2
4. All OOH-physicians take risks; it’s risk assessment OOH all the time (n = 99)	17	29	21	31	1	3.3
5. OOH-physicians are good at living with uncertainty and risk	9	48	31	11	1	3.5
6. I don’t worry about my decisions after I’ve made them	8	42	4	41	5	3.1
7. I sometimes go back and check on the patient’s outcome after a shift has finished	10	41	12	26	11	3.1

Five-point Likert scale (n = 100, unless otherwise stated).

(*Dimension A of the questionnaire. The seven items were used to create the Tolerance of Risk scale).

The other three dimensions (B-D) concerned chest pain patients only. Dimensions B – D measured attitudes to hospital admission, including patient related and relative related influence on decision making.

In dimension B, we found that half of the physicians (51%, mean 3.0) worry about complaints being made about them, but few let fear of complaints from the Board of Health Supervision influence their practice (16%, mean 2.1).

Dimension C examined attitudes to hospital admission. 69% (mean 3.6) agreed that admitting someone to hospital enables them to get a second opinion, but 75% (mean 3.7) also agreed that admitting someone to hospital put patients in danger of being “over-tested”.

The last dimension (D) concerned patient-related factors. There was a strong agreement that the patient’s clinical status was the most important factor (96% agreed, mean 4.6) in deciding to admit a patient or not. Half of the physicians were more likely to admit the patient if the patient himself wanted to be admitted (51% agreed, mean 3.2), or if a family member wanted the patient to be admitted (46% agreed, mean 3.1).

Overall mean scores from all items in the four dimensions were also compared with mean scores within the three risk groups derived from the Pearson Risk Scale. In dimension A, concerning all patients out-of-hours, there is a clear trend in most items that the “risk avoiders” differ from the rest, and there is a significant difference in the statement “When it comes to OOH-medicine I’m quite cautious” (p = 0.024). In dimension B, we found a significant difference in the statement “I don’t worry about a complaint being made about me” (p = 0.006), where the group “risk avoiders” had a mean score of 2.2 versus the mean score of 3.2 for the rest of the physicians. There were no significant differences when testing the “risk avoiders” against the rest in each of the five items in dimension C. In the last dimension (D), we found significant differences in the statements “I am more likely to admit a person if they want to be admitted” (p = 0.039), “If members of the family say there’s nobody to look after someone, I see that as a problem for the family rather than the doctor” (p = 0.034) and “I am more likely to admit someone if they live alone” (p = 0.008).

## Discussion

“Patient history and symptoms” was by far the most important aspect in the diagnostic process, while “negative ECG” and “effect of sublingual nitroglycerine” was considered least important. We found no significant differences in length of experience or gender when testing “risk avoiders” (neither Pearson Risk Scale nor Tolerance of Risk Scale) against the rest. Almost all physicians felt that their risk assessment out-of-hours was reasonably good, and felt reasonably safe, but only half of them agreed with the statement “I don’t worry about my decisions after I’ve made them”. Concerning chest pain patients only (dimension B-D), about half of the physicians worried about complaints being made about them, the vast majority agreed that admitting someone to hospital put patients in danger of being “over-tested”, and about half of the physicians were more likely to admit the patient if they wanted to be admitted.

Main strengths of the study include the prospective study design with the use of telephone interviews shortly after a consultation, to gather data. This allowed the interviewer to give precise instructions and guidance. Some of the questions concerned the patient they recently had treated, and we aimed to reduce recall bias by reaching the physician shortly after the consultation (with a maximum of 2 days). An important limitation of the study is the number of included patients and physicians (n = 100), because of limited resources available for interviews.

Ruling out or confirming acute ischaemic heart disease (IHD) is widely considered the most important aspect when dealing with chest pain outside hospitals. A meta-analysis from 2008 on the accuracy of symptoms and signs in diagnosing coronary heart disease [5] confirmed that patient history with symptoms is clinically important, but no symptom itself had a major impact on the post-test probability of IHD in a low-prevalence setting (i.e. general practice). However, the presence of chest-wall tenderness largely ruled out IHD, with a post-test probability of only 1%. Similar results were found by Bösner et al. in 2010 [6]. Recently published guidelines from the British National Institute for Health and Care Excellence (NICE) concerning chest pain of recent onset recommend that physicians should not use the patient’s response to sublingual nitroglycerine when diagnosing patients with chest pain [16]. Extensive research has shown that ECG is a diagnostic tool with relatively high specificity, but with limited sensitivity [17,18] and physicians should be careful ruling out IHD on the basis of a normal resting ECG alone. Our study showed that almost all physicians regarded a patient’s symptoms/history and possible “positive ECG”-findings as fairly or very important in the diagnostic approach. These results concur with current evidence. The vast majority also adjudged “negative ECG”-findings to be less important, but almost a fourth considered negative findings to be important. As many as 40% believed that the effect of nitroglycerine was important and over half believed that the presence of chest-wall tenderness was of little importance. A research group in Germany has recently developed and externally validated a clinical decision rule for ruling out coronary heart disease in primary care (Marburg Heart Score) [19,20]. The Marburg Heart Score has shown promising results, and might lead to a breakthrough in the use of clinical decision rules in patients with chest pain outside hospitals.

The parts of our questionnaire containing four dimensions on “tolerance of risk” and “attitudes to hospital admission” were derived from a questionnaire previously published in an article by Ingram et al. in 2009 [12]. A main finding from that study was that GPs with “low tolerance of risk” and female GPs were more likely to refer patients to the hospital out-of-hours, but the female GPs referred more because they were more inclined to be “risk averse”. In 2007, Rossdale et al. also found that female GPs referred more patients out-of-hours than their male counterparts, and that length of work experience as GP did not influence referral rates [13]. Calnan et al. found in a qualitative study that high referring GPs out-of-hours typically are more cautious and would admit more often if in doubt [14].

Pearson et al. developed the “Risk-taking Scale” in 1995 for use in triage decisions for emergency department patients with chest pain [15]. They found that physician risk attitudes correlated significantly with admission rates for patients with acute chest pain. The “risk-seeking” physicians admitted only 31% of the patients with chest pain, compared with 53% for the physicians with low risk–taking scores (“risk-avoiders”).

Our study did not have a design that allowed comparison between “tolerance of risk” and referral/admission rates. However, we did show that physicians vary in their “tolerance of risk” in out-of-hours work. This variation was not dependent on gender or length of experience. We also showed that physicians vary considerably in what influences their decision to admit a patient with chest pain to a hospital or not.

The differences in diagnostic approach found in our study highlight the need for continuous education of GPs on diagnosing chest pain in primary care. A recently published article from another part of our study also revealed the challenges in management of chest pain outside hospitals [11]. Most patients were investigated for ischaemic heart disease, but less than half were admitted to hospital for suspected heart disease, and few were actually given emergency treatment for acute coronary syndrome at the casualty clinics [11]. This sheds light on the fact that patients with chest pain in primary care most often do not suffer from acute ischaemic heart disease. Focus should be more on diagnosing the probable cause, with appropriate management, and less on “ruling out” ischaemic heart disease alone.

Our findings on “tolerance of risk” and “reasons for hospital admission” also support the need for educational programmes to empower primary care physicians on decision-making and confidence. It is well known that physicians vary considerably in attitude and confidence. However, we believe that specific education on risk-stratification and pre-test probabilities of important medical conditions, in different settings, will contribute to the right decision being made, with less influence from the physicians’ attitude and tolerance of risk. Continuous medical education should also to a greater extent focus on what influence the physicians’ risk assessment out-of-hours and decisions on treatment and right level of care. In countries where primary care physicians function as “gatekeepers”, like Norway, empowerment of the physicians through training and focus on “tolerance of risk”, will probably lead to more appropriate referrals and better management of patients out-of-hours.

## Conclusions

Physicians working out-of-hours showed considerable differences in their diagnostic approach, and not all physicians diagnose patients with chest pain according to current guidelines and evidence. Differences in “tolerance of risk” have a substantial influence on how physicians decide to manage patients with chest pain out-of-hours, and the physicians vary considerably in what may influence their decision to admit a patient with chest pain to a hospital or not. Continuous medical education must focus on the diagnostic approach in patients with chest pain in primary care and empowerment of physicians through training and emphasis on risk assessment and “tolerance of risk”.

### Consent

Written informed consent was not obtained from the patients for this paper because in all collected data the patients were anonymous. This was approved by the Regional Committee for Medical and Health Research Ethics (REC West) before inclusion started.

## Abbreviations

ECG: Electrocardiography
GP: General practitioner
OOH: Out-of-hours
IHD: Ischaemic heart disease
